# Intragenomic conflict produces sex ratio dynamics that favor maternal sex ratio distorters

**DOI:** 10.1002/ece3.2498

**Published:** 2016-10-15

**Authors:** Elaine S. Rood, Steven Freedberg

**Affiliations:** ^1^ Department of BiologySt. Olaf CollegeNorthfieldMNUSA

**Keywords:** genetic conflict, population sex ratio, population‐level selection, sex ratio distorter

## Abstract

Maternal sex ratio distorters (MSDs) are selfish elements that enhance their transmission by biasing their host's sex allocation in favor of females. While previous models have predicted that the female‐biased populations resulting from sex ratio distortion can benefit from enhanced productivity, these models neglect Fisherian selection for nuclear suppressors, an unrealistic assumption in most systems. We used individual‐based computer simulation modeling to explore the intragenomic conflict between sex ratio distorters and their suppressors and explored the impacts of these dynamics on population‐level competition between species characterized by MSDs and those lacking them. The conflict between distorters and suppressors was capable of producing large cyclical fluctuations in the population sex ratio and reproductive rate. Despite fitness costs associated with the distorters and suppressors, MSD populations often exhibited enhanced productivity and outcompeted non‐MSD populations in single and multiple‐population competition simulations. Notably, the conflict itself is beneficial to the success of populations, as sex ratio oscillations limit the competitive deficits associated with prolonged periods of male rarity. Although intragenomic conflict has been historically viewed as deleterious to populations, our results suggest that distorter–suppressor conflict can provide population‐level advantages, potentially helping to explain the persistence of sex ratio distorters in a range of taxa.

## 1 Introduction

Population sex ratios should be even at equilibrium due to frequency‐dependent selection for the rarer sex (Düsing, 1884; Fisher, 1930). Uniparental sex factors can cause strong deviations from even sex ratios, with selection favoring elements that bias the sex ratio toward the transmitting sex, increasing the transmission rate of the sex factor. These sex factors are commonly manifested as maternally transmitted parasites or cytoplasmic alleles that feminize the sex ratio by altering the mechanistic control over sexual differentiation or killing male embryos (reviewed in Hurst, 1993; Engelstadter & Hurst, 2009). Maternal sex ratio distorters (MSDs) have been documented in a wide range of dioecious species and are particularly well represented in arthropods and plants (Jiggins, Bentley, Majerus, & Hurst, 2001; Taylor & Ingvarsson, 2003). Sex ratio distorter systems have been documented to possess single genetic or parasitic distorters, multiple parasitic sex ratio distorters, or a combination of genetic and parasitic factors (Kageyama, Nishimura, Hoshizaki, & Ishikawa, 2003; Rigaud & Juchault, 1993).

Female‐biased population sex ratios associated with MSDs are predicted to result in Fisherian selection favoring the production of males. In many MSD systems, nuclear suppressor loci have been identified that are capable of eliminating or reducing the feminizing effects associated with distorters (Hornett et al., 2006, 2014; Majerus & Majerus, 2010; Rigaud & Juchault, 1993). The countervailing selective pressures between Fisherian selection and cytoplasmic sex ratio distorters are predicted to set off a dynamic conflict between distorters and their suppressors (Hatcher, Taneyhill, Dunn, & Tofts, 1999; Hurst, 1993; Werren, 1987), potentially resulting in temporal fluctuations in population sex ratios (Hornett, Charlat, Wedell, Jiggins, & Hurst, 2009; Randerson, Smith, & Hurst, 2000).

Intragenomic conflict is generally viewed as a process involving elements that enhance their transmission at the expense of individual or population success (Hatcher, Dunn, & Tofts, 2000). In addition, strong population sex ratio biases associated with MSDs can lead to population extinction via male rarity and the Allee effect (Hatcher et al., 1999). Skewed population sex ratios are also predicted to be accompanied by reductions in effective population size (*N*_*e*_, (Wright, 1931; Engelstadter & Hurst, 2007), further increasing the risk of extinction (Newman & Pilson, 1997). Beyond the costs associated with altered sex allocation, sex ratio distorters and their suppressors are frequently characterized by pleiotropic fitness reduction (Atlan, Joly, Capillon, & Montchamp‐Moreau, 2004; Price et al., 2008; Wu, True, & Johnson, 1989). Despite these well‐documented costs, species possessing sex ratio distorters are well‐represented taxonomically. The commonality of systems characterized by elevated likelihood of population extinction suggests they are offset by population‐level advantages (Williams, 1992).

Reproductive productivity associated with biased sex ratios has been implicated in the evolutionary dynamics of sex ratio distorter systems (Hamilton, 1967). Werren and Beukeboom (1993) found that a male‐biasing chromosome in *Nasonia vitripennis* reduced the intrinsic rate of increase within a meta‐population and predicted that moderate levels of female bias may provide a competitive advantage for populations due to an increased reproductive rate. James and Jaenike (1990) speculated that female biases owing to X chromosome meiotic drive in *Drosophila testacea* may yield advantages via reduced sexual selection and enhanced interspecific competition for larval food sources. Sex ratio distorters have been implicated in the evolutionary success of invasive species (Galbreath, Smith, Terry, Becnel, & Dunn, 2004), a trend attributable to an enhanced rate of population increase (Hatcher et al., 1999). Recently, Unckless and Clark (2014) modeled the impacts of female‐biased sex ratios resulting from sex ratio meiotic drive on the competitive ability of species in a *Drosophila* community. Although the model developed by Unckless and Clark highlights the logical conclusion that female‐biased sex ratios will increase reproductive rate, it fails to incorporate the other half of the dynamic, selection for nuclear suppressors. While rare exceptions exist, suppressors appear to be common in most MSD systems (Taylor & Ingvarsson, 2003) and are predicted to rapidly reduce or eliminate population sex ratio biases. Thus, the sex ratio dynamics of real MSD systems can only be explored when the interplay between both distorters and their suppressors is considered.

In this article, we take a computer simulation‐based approach to study maternal sex ratio distorter–suppressor conflict and its role on interspecific interpopulation competition. We examine sex ratio dynamics in the presence of mutations causing cytoplasmic sex ratio distortion and their nuclear suppressors. We explore the competitiveness of populations characterized by these distorter–suppressor systems under both single population colonization and multiple‐population scenarios. Furthermore, we assess the impact of variation in distorter number and fitness costs associated with distortion and suppression. The results of this study may shed light on the factors contributing to the commonality of systems characterized by selfish sex ratio distorters.

## 2 Methods

### 2.1 Model

We considered populations of sexual diploid individuals in a polygynous mating system. Mating was random, and the population was regulated via a carrying capacity (*K)*. The reproductive rate (*R*) each generation equaled the carrying capacity divided by the total population size, with a maximum *R* of 20. Each generation, the total number of reproductive events was 2R*(# of females), and females were randomly chosen to reproduce. Breeding was semelparous, and generations were nonoverlapping. Populations began at carrying capacity unless otherwise noted (see Scenarios).

There were two types of species: one that could receive a distorter mutation capable of biasing the brood sex ratio of affected females, and the other unable to receive the distorting mutation. Every generation, each individual in the distorting species could receive a maternally inherited (cytoplasmic) mutation, with probability α, that caused mothers to produce a distorted sex ratio. Distorter cytotypes could mutate back to wild type with the same probability. The distorter was transmitted from mothers to daughters with a rate of transmission β. The distorting cytotype caused mothers to produce an assigned proportion γ females, where 0.5 ≤ γ ≤ 1. Distorter females suffered a discrete reduction in fecundity δ, manifested as the probability that a female failed to produce an offspring in a given reproductive event.

All females suffered a cost of reduced fertilization when males were rare, applied to the number of offspring produced. The cost manifested as a reduction (ε) in offspring production when the population sex ratio (proportion female, *F*) was female biased:

$$\epsilon =625\times {\left(F-0.5\right)}^{10}.$$

This equation produces a reduction in fecundity identical to that observed in the best‐studied population characterized by a prolonged extremely female‐biased sex ratio resulting from a MSD (Dyson & Hurst, 2004). *R* was set to zero when males were completely absent from the population, causing the population to immediately go extinct.

The sex‐determining effects of the distorter could be counteracted by an autosomal dominant “suppressor” allele (Hornett et al., 2006; Mercot, Atlan, Jacques, & Montchampmoreau, 1995; Rigaud, Moreau, & Juchault, 1999; Tao, Masly, Araripe, Ke, & Hartl, 2007) that returned γ to 0.5 for a female possessing the corresponding the distorter. This allele arose from a random mutation with probability θ, followed Mendelian inheritance, and could mutate back to the wild‐type allele with the same probability. The suppressor had a fitness cost λ (Wu et al., 1989) that represented the probability that a female possessing the allele failed to produce an offspring in a given reproductive event. Individuals possessing both the distorter and suppressor suffered additive fitness costs associated with the suppressor and distorter. The suppressor allele exhibited the same “silent” cost in the absence of the distorter (Wu et al., 1989).

In a subset of our simulations, two different distorting cytotypes could occur, each of which was capable of producing a proportion γ females. Each distorter had a corresponding suppressor locus, with each suppressor allele at these loci capable of restoring the offspring sex ratio to 0.5 only for its corresponding cytotype. Cytoplasmic mutations could cause shifts between the wild cytotype and two distorting cytotypes, as well as between the two distorting cytotypes at a frequency α.

### 2.2 Simulations

We ran the given number of simulations across every percentage of distorter sex ratio (from 50% to 100% female) for each set of parameters under the following scenarios:

1. Single population competition. After a 500 generation inoculation, a population was invaded by 10 individuals from the opposing species. Interspecific competition was incorporated by instituting a carrying capacity (*K*) for the combined population size of the resident and colonizing individuals. We ran 500 simulations at each level of distorter sex ratio for each combination of resident/colonizer (MSD invading non‐MSD and vice versa), for a total of 25,500 simulations. Simulations were run until one species was eliminated from the simulation.
2. Multiple‐population competition. Nine separate “habitats” existed, where distorting and nondistorting individuals competed as above. At the start of each simulation, three habitats each contained *K* individuals of the distorting species, three habitats contained *K* individuals from the nondistorting species, and three areas were unoccupied, but could eventually be filled by migrants of either species. After an equilibration period of 500 generations, migration was allowed at a rate μ, and simulations were run until one species was absent from all nine habitats. We ran 250 simulations at each level of distorter sex ratio for a total of 12,750 simulations.

The default parameters for all scenarios were *K* = 1,000, α = 0.001, β = 1, θ = 0.00005, δ = 0.1, λ = 0.1. In two‐distorter simulations, each distorter and suppressor exhibited identical fitness costs and sex ratio biases. We examined the impacts of variation in fitness costs (δ, λ) and distorter sex ratio (γ) on allele frequencies, population sex ratios and the relative frequencies of MSD and non‐MSD individuals. Fitness costs for distorters and suppressors could take on values of 5% (low), 10% (moderate), or 20% (high).

When migration was examined, the default emigration rate μ per individual from a given population was 0.002, with an equal probability of migrating into each of the other eight populations. Both distorting and nondistorting species were assumed to have the same migration rates. All simulations were run in Perl 5.16. Statistical analyses were run in R 3.2.3. Source code is available from SF upon request.

## 3 Results

### 3.1 Sex ratio dynamics

#### 3.1.1 One‐distorter simulations

**Figure 1:**
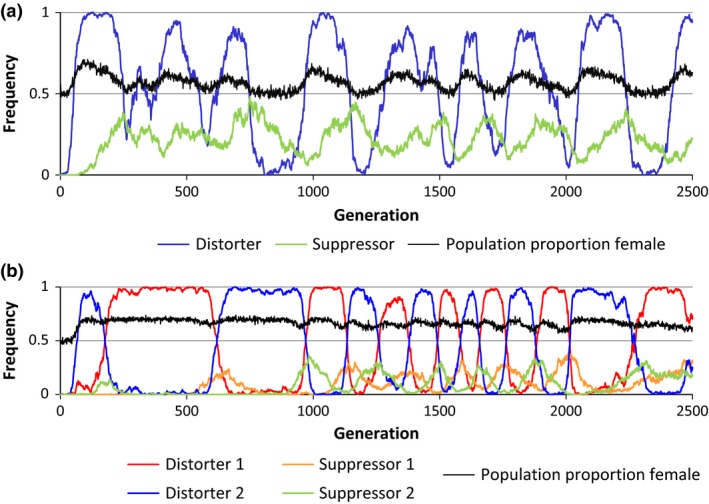
Population sex ratio associated with distorter and suppressor allele dynamics. In one‐distorter simulations, population sex ratios fluctuated between female biased when the distorting cytotype was common and 1:1 when the wild cytotype was common (a). In two‐distorter simulations, the alternating relationship between the two distorting cytotypes allowed female biases to be maintained consistently throughout the simulations (b). Distorter cost (δ) = 0.2; suppressor cost (λ) = 0.05

In all simulations, cytotypes carrying a female‐biasing distorter rapidly increased in frequency, resulting in a sharp increase in the sex ratio (proportion female). Under medium and low suppressor costs, the suppressor allele increased in frequency after the distorter became common, reducing the sex ratio bias. Suppressors bearing high costs invaded only when the population sex ratio became highly skewed. When assuming that distorter systems possess low suppressor and high distorter fitness costs (Hurst & Pomiankowski, 1991), a cyclical dynamic appeared between the distorter and suppressor alleles. Specifically, the spread of the suppressor was followed by a loss of the distorter allele and then the loss of the suppressor allele. After the suppressor allele became rare, the wild cytotype spread, followed by the spread of the distorter (Figure 1a). This cyclical exchange between the distorter and wild cytotype resulted in the population sex ratio fluctuating between female biased and even. At high suppressor costs and moderate‐to‐small sex ratio biases, suppressor alleles failed to increase above low frequencies (i.e., <5%), allowing the female bias to be maintained throughout the simulation.

#### 3.1.2 Two‐distorter simulations

Under all levels of sex ratio distortion, the cytotype alternated between the two distorter types, with the wild cytotype maintaining a low frequency throughout the simulation (<5%). As in one‐distorter simulations, the first distorter mutation to appear spread rapidly, which was followed by the spread of its specific suppressor allele. Following the spread of the suppressor allele, the first distorting cytotype decreased in frequency, coinciding with the spread of the alternate distorter and then its suppressor (Figure 1b). This cycle was maintained throughout the simulation and occurred at a wide range of distorter costs and sex ratios. In contrast to the one‐distorter simulations, population sex ratios rarely returned to even, owing to the near constant presence of one of the two distorting cytotypes. Similar to one‐distorter simulations, high suppressor costs prevented the spread of the suppressor in two‐distorter simulations, and the dominant cytotype fluctuated stochastically between the two distorting cytotypes.

### 3.2 Single population competition

In both one‐distorter and two‐distorter simulations, invasion success was strongly influenced by the offspring sex ratio produced by the distorter, with intermediate levels of sex ratio bias resulting in the strongest competitive advantage for the distorting populations. Under the default parameters of fitness costs, the distorting population was significantly more likely to competitively displace a nondistorting population than was a comparable invading nondistorting population at all levels of sex ratio bias from 56% to 84% females for the one‐distorter model and from 56% to 89% for the two‐distorter model (two‐sample proportion test; *p* < .01 for all analyses). Post hoc analysis revealed that populations with distorters producing large sex ratio biases (>90% female) primarily failed to invade owing to high suppressor frequencies producing unbiased population sex ratios in distorting populations.

**Figure 2:**
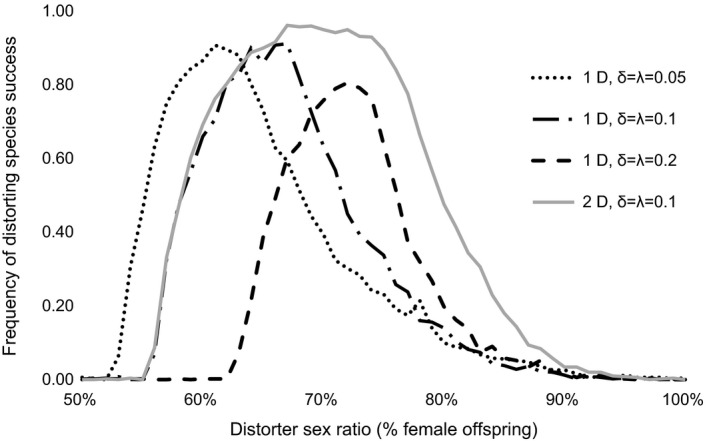
Invasion success of an MSD population invading a nondistorting population in the single population scenario. Distorting populations varied in number of distorters (1D vs 2D) and distorter (δ) and suppressor (λ) costs

Invasion success decreased with increase in distorter cost and increased with increase in suppressor cost. High distorter fitness costs (δ = 0.2) resulted in invasion success at a relatively narrow range of distorter sex ratios (Figure 2). The higher invasion success associated with high suppressor costs can likely be attributed to low suppressor allele frequencies, which allowed the sex ratio of distorting populations to remain female biased for longer periods.

Two‐distorter simulations yielded invasion probabilities that were equal to or greater than invasion success for one‐distorter simulations for all distorter sex ratios tested. For one‐distorter simulations, invasion success was limited at higher distorter sex ratios due to the increase in suppressors associated with these distorters. Conversely, two‐distorter populations were more successful at high distorter sex ratios owing to the fact that suppression of the first distorter was followed by invasion of the second distorting cytotype.

Nondistorting populations failed to displace resident distorting populations at low and moderate levels of sex ratio distortion in the distorting populations. Resident distorting populations were more likely than resident nondistorting population to be competitively displaced by invading nondistorting populations at sex ratios above 77% for one‐distorter simulations and above 85% for two‐distorter simulations (two‐sample proportion test; *p* < .01 for all analyses).

### 3.3 Multiple‐population competition

**Figure 3:**
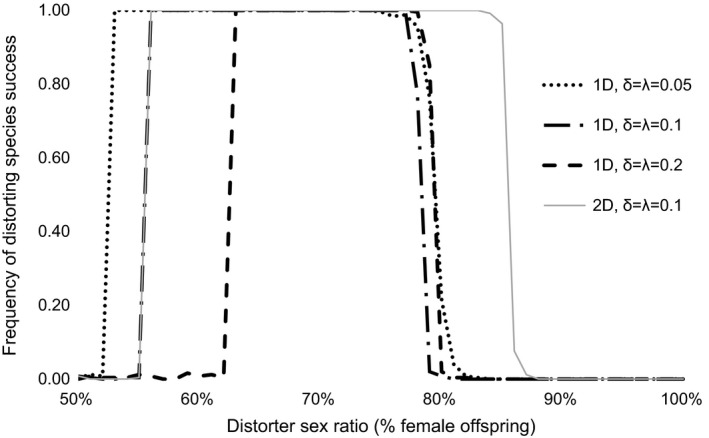
Establishment success of an MSD population competing against a nondistorting population in the multiple‐population scenario. Distorting populations varied in distorter (δ) and suppressor (λ) costs

**Figure 4:**
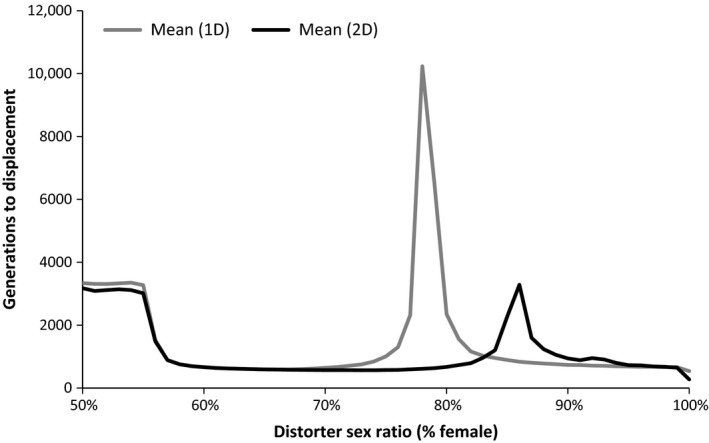
Time (generations) to displacement in multiple‐population simulations for one‐distorter and two‐distorter simulations. Competition was initiated after a 500 generation inoculation period

The level of sex ratio distortion had a significant effect on establishment in the multiple‐population model, with distorting populations becoming established at a higher rate than nondistorting populations at all levels of sex ratio bias from 56% to 78% female for one‐distorter and from 56% to 85% female for two‐distorter simulations (two‐sample proportion test; *p* < .01 for all analyses). The width of the range of distorter sex ratios at which distorting populations successfully invaded nondistorting populations decreased with increase in distorter costs and increased with increase in suppressor costs (Figure 3). Two‐distorter simulations yielded establishment probabilities that were equal to or higher than establishment success for one‐distorter simulations for all distorter sex ratios tested. The time to displacement (number of generations required to allow one species to be excluded from all nine habitats) was influenced by the number of distorters, as one‐distorter simulations took longer to reach displacement than two‐distorter simulations (two‐sample proportion test, *p* < .0001; Figure 4).

## 4 Discussion

We found that the co‐occurrence of maternal sex ratio distorters and nuclear suppressors resulted in periodic or persistent female‐biased population sex ratios, and these dynamics were affected by the degree of distorter sex ratio bias and the fitness costs associated with distorter and suppressor alleles. Invading MSD populations generally experienced elevated reproductive output and establishment when competing with resident populations lacking sex ratio distorters. When competing over a range of interconnected habitats, distorting species frequently displaced nondistorting species. Our findings suggest that the sex ratio dynamics resulting from genomic conflict between maternally inherited sex ratio distorters and their suppressors can, under a fairly broad range of conditions, favor species characterized by sex ratio distorters over those lacking them. Given the importance of interspecific competition in determining evolutionary success and range limits (Duyck, David, & Quilici, 2004; Price & Kirkpatrick, 2009), our findings may reveal an important mechanism contributing to the distribution of species possessing sex ratio distorters.

Our results were highly dependent on the levels of sex ratio distortion and distorter and suppressor costs, values that have been found to vary both between and within species. The parameter ranges that produced an advantage for MSD species in our simulations overlap with those quantified from many empirical studies. Genetic cytoplasmic distorters normally exhibit perfect maternal vertical transmission, and near‐perfect or perfect transmission rates near have been documented in parasitic systems as well (Charlat et al., 2009; Jiggins, Randerson, Hurst, & Majerus, 2002). While some distorters are capable of completely feminizing broods, many distorters have more modest impacts, and distorter brood sex ratios frequently range from 60% to 80% female (Hassan, Idris, & Majerus, 2012; Mercot et al., 1995; Rigaud et al., 1999; Vala, Van Opijnen, Breeuwer, & Sabelis, 2003). Pleiotropic fitness costs associated with distorters are frequently manifested as a reduction in egg production (Kelly, Dunn, & Hatcher, 2001; Rigaud et al., 1999). Many distorters are accompanied by small (<20%) reductions in fecundity, a range yielding population‐level advantages in our simulations (Hoffmann, Turelli, & Harshman, 1990; Kelly et al., 2001; Rigaud et al., 1999). We found that biased population sex ratios can be maintained when nuclear suppressors exhibit nonzero fitness costs, preventing the suppressors from spreading to fixation. Fitness reductions associated with suppressors have been documented in both dioecious and gynodioecious systems and occur even in the absence of the associated distorter (Bailey, 2002; Wu et al., 1989). Suppressor costs have been further implicated in MSD species by the observation that frequencies of suppressor alleles are lower than predicted by Fisherian selection alone (Hornett et al., 2014; Vaz & Carvalho, 2004).

**Figure 5:**
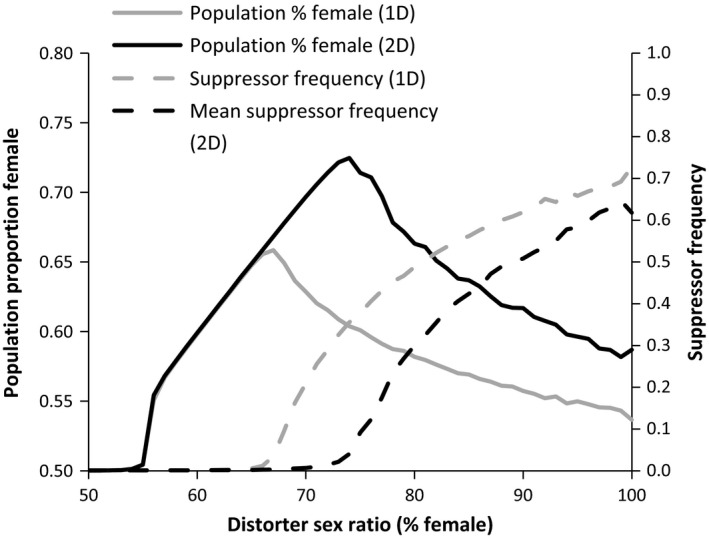
The effects of distorter sex ratios on average population sex ratios and suppressor frequencies. For two‐distorter simulations, suppressor frequency represents the average of both alleles

Across all parameters examined, populations with distorters producing moderate female biases exhibited greater competitive ability than nondistorting populations, while populations with distorters producing very weak or very strong sex ratio biases were at a competitive disadvantage when paired with nondistorting populations. The reduced competitive ability of populations with weak distorters can be attributed to distorter and suppressor fitness costs that outweighed the modest increase in productivity associated with a small female bias. The lack of a competitive advantage in populations characterized by strong distorters was due to the spread of suppressors, maintaining the sex ratio near even (Figure 5). Very strong distorters (~100% female) were associated with low population productivity and elevated extinction rates resulting from male rarity (Hatcher et al., 1999).

While it has previously been proposed that the presence of a female‐biasing force can increase the productivity of groups or populations (Hamilton, 1967; Unckless & Clark, 2014), sex ratio distorters are predicted to be countered by suppressing elements, and thus, it is essential to consider the impacts of these suppressors on population dynamics. We found that conditions leading to the fixation of suppressors eliminated the enhanced productivity associated with distorters. In most simulations, suppressors failed to spread to fixation, and the presence of moderate levels of suppression reduced the probability of demographic population extinction in populations with strong distorters. Furthermore, although the benefits associated with female‐biased sex ratios may be diminished by the reduction in genetic diversity in populations with skewed sex ratios (Engelstadter & Hurst, 2007), the repeated restoration of unbiased sex ratios associated with the spread of suppressors mitigates these effects, as populations with occasional expansions in population size experience an elevated *N*_*e*_ relative to those with consistently small population size (Kalinowski & Waples, 2002). For instance, a population with a consistent 2:1 sex ratio experiences an 11.2% reduction in *N*_*e*_ relative to an unbiased population, whereas a population that oscillates between 2:1 and 1:1, while still capable of competitively excluding unbiased populations, experiences only a 5.9% reduction in *N*_*e*_.

The nature of the dynamic between distorters and suppressors was strongly mediated by their fitness costs. Neutrality of distorter and suppressor alleles resulted in fixation of both, producing even sex ratios, consistent with earlier predictions (Vaz & Carvalho, 2004). High suppressor costs prevented the spread of the suppressor, allowing a sex ratio bias to persist throughout the simulation. When we modeled a single distorter with costs that exceeded suppressor costs, as has previously been assumed (Hurst & Pomiankowski, 1991), a cyclical dynamic was observed between the wild cytotype, distorting cytotype, and suppressor (Figure 1a). This pattern and resulting sex ratio fluctuations match those observed over time in *Hypolimnas* butterflies infected by cytoplasmically transmitted male‐killing *Wolbachia* (Hornett et al., 2009). In two‐distorter simulations with low suppressor costs, the first distorting cytotype was lost after the spread of its suppressor, immediately followed by the invasion of the second distorting cytotype, mirroring the pattern predicted by Randerson et al. (2000). Subsequent alternating cycles of the two distorting cytotypes prevented the spread of the wild cytotype, allowing a consistent female bias to be maintained over time (Figure 1b).

While one‐ and two‐distorter populations exhibited comparable competitive ability for moderately weak sex ratio distorters (<65% females), two‐distorter populations experienced greater competitive advantages at higher levels of sex ratio distortion (Figures 2 and 3). Post hoc analyses revealed that the frequency of suppressors began increasing at lower levels of population sex ratio bias in one‐distorter simulations relative to two‐distorter simulations (Figure 4). In one‐distorter simulations, the presence of the distorting cytotype resulted in strong directional selection favoring the suppressor allele. In two‐distorter systems, the distorting cytotype initially resulted in selection for its unique suppressor, but the spread of the second distorting cytotype then removed any Fisherian benefits of the first suppressor. Because male‐production required suppressors (and their associated fitness costs) to be present at two loci, suppressors spread to appreciable frequencies only after strongly biased population sex ratios were present.

Species possessing MSDs can exhibit substantial among‐population variation in population sex ratios (Hatcher et al., 2000; Hornett et al., 2009; Taylor, 1999) and the resulting mosaic of competitive abilities between MSD and non‐MSD species may prevent one species type from excluding the other over a range of habitats. In our multiple‐population one‐distorter simulations, some parameter sets allowed distorting and nondistorting populations to coexist for thousands of generations (Figure 4). The cyclical pattern of female biases in one‐distorter systems (Figure 1a) was asynchronous among populations, allowing MSD populations to displace nondistorting populations in habitats encountered at a time when the MSD population was female‐biased, while nondistorting populations persisted in habitats where the MSD population had an unbiased sex ratio. Migration from successful habitats then allowed extirpated cytotypes to continually be re‐inoculated. This pattern is analogous to that predicted by models showing a stable polymorphism of distorters and nondistorters in structured populations via re‐colonization following local extinction (Hatcher et al., 2000). However, extinction in those models is attributed to male rarity in MSD populations, whereas extinction in our simulations generally resulted from competitive exclusion of non‐MSD populations by more productive MSD populations. In contrast to one‐distorter simulations, the sex ratio in two‐distorter simulations was relatively consistent across all populations, resulting in rapid displacement of the species that was at a competitive disadvantage.

### 4.1 Sex ratio biases and higher level selection

Individual selection is predicted to favor even sex ratios in large populations regardless of potential benefits of biased sex ratios to population‐level productivity (Hamilton, 1967). While advantages to populations cannot override Fisherian selection to produce female‐biased allocation, nonadaptive phenomena such as intragenomic conflict and infection by maternally inherited parasites can allow sex ratio biases to occur in some populations. This variation in population sex ratios, even when transitory, can be acted upon by selection if it affects the productivity or survival of populations. Our results reveal that population sex ratio fluctuations that are a byproduct of nuclear‐cytoplasmic conflict can provide a competitive advantage to populations, allowing populations possessing MSDs to displace populations with even sex ratios.

In order to assign higher levels of selection, it is critical to ask where the locus of selection lies. For example, some instances of apparent higher‐level selection can, in fact, be attributed to the accumulated action of individual selection if the pattern results from aggregate instances of differential productivity of individual organisms (Folse & Roughgarden, 2010). Claims of selection above the individual organism often focus on groups of individuals within a population; however, selection can favor entire populations that exhibit elevated productivity or survival (Tellier, Villareal, & Giraud, 2007). If groups display differential productivity irrespective of the success of their constituents, such as populations successfully colonizing daughter populations, higher‐level selection can be invoked (Damuth & Heisler, 1988; Okasha, 2006). In our multiple‐population simulations, MSD populations had lower extinction rates and higher proliferation rates than non‐MSD populations, attributes reflecting the “emergent fitness” of populations (Folse & Roughgarden, 2010). This pattern cannot be explained by the accumulated effects of individual selection, as the fitness of individuals was optimized by even investment in the sexes.

Although generally viewed as an intraspecific process (Aviles, 1993), selection on female production can play an important role in competition between species. By avoiding the cost of males, asexual species experience higher reproductive rates, allowing them to potentially outcompete their sexual counterparts for shared resources (Case & Taper, 1986). Species with environmental sex determination (ESD) are more prone to produce female‐biased populations than species with genetic sex determination, offering a colonization advantage that can contribute to the persistence and success of many ESD taxa (Freedberg & Taylor, 2007). Female‐biased sex ratios associated with X‐linked meiotic drive impart an advantage that may shape the community composition of competing *Drosophila* species (Unckless & Clark, 2014). While our results can help to explain why MSD systems greatly outnumber systems with paternal sex ratio distorters (PSDs), this pattern may also be attributed to a greater prevalence of maternally inherited transmission agents. We predict that the enhanced population‐level competitive ability will result in longer persistence of MSD relative to PSD species and encourage attempts aimed at documenting these patterns in natural populations (Atlan, Mercot, Landre, & Montchamp‐Moreau, 1997).

Are traits arising from intraindividual conflict more likely to contribute to higher levels of selection? Among‐population selection is predicated on variation among populations in traits that may differentially affect their colonization or extinction rates. Competing individual organisms will tend to have closely aligned fitness optima, which are reached via maximizing reproductive opportunities and the acquisition of resources. Consequently, individual selection should contribute relatively modestly to among‐population trait variation. Conversely, selfish elements with different transmission patterns have opposing fitness optima, and conflict can result in rapid shifts between these peaks (Bastide, Gerard, Ogereau, Cazemajor, & Montchamp‐Moreau, 2013). Interacting populations that are situated at different points in the adaptive landscape will differ significantly in traits related to the transmission of these elements, and these traits may contribute to the competitive ability of populations. While we found that temporal cycling of sex ratios resulting from MSD–suppressor conflict can produce substantial variation in population‐level fitness, future studies on the population dynamics associated with other forms of genomic conflict can shed light on the relationship between selection at different levels of organization.

## Conflict of Interest

None declared.
